# Eco-Friendly Triboelectric Material Based on Natural Rubber and Activated Carbon from Human Hair

**DOI:** 10.3390/polym14061110

**Published:** 2022-03-10

**Authors:** Tanapon Chomjun, Intuorn Appamato, Viyada Harnchana, Vittaya Amornkitbamrung

**Affiliations:** 1 Department of Physics, Khon Kaen University, Khon Kaen 40002, Thailand; tanapon.chj@gmail.com (T.C.); vittaya@kku.ac.th (V.A.); 2 Materials Science and Nanotechnology Program, Faculty of Science, Khon Kaen University, Khon Kaen 40002, Thailand; intuorn.ap.95@gmail.com; 3 Institute of Nanomaterials Research and Innovation for Energy (IN-RIE), Khon Kaen University, Khon Kaen 40002, Thailand

**Keywords:** triboelectric nanogenerator, natural rubber, activated carbon, human hair

## Abstract

The triboelectric nanogenerator (TENG) has emerged as a novel energy technology that converts mechanical energy from surrounding environments to electricity. The TENG fabricated from environmentally friendly materials would encourage the development of next-generation energy technologies that are green and sustainable. In the present work, a green triboelectric material has been fabricated from natural rubber (NR) filled with activated carbon (AC) derived from human hair. It is found that the TENG fabricated from an NR-AC composite as a tribopositive material and a poly-tetrafluoroethylene (PTFE) sheet as a tribonegative one generates the highest peak-to-peak output voltage of 89.6 V, highest peak-to-peak output current of 6.9 µA, and can deliver the maximum power density of 242 mW/m^2^. The finding of this work presents a potential solution for the development of a green and sustainable energy source.

## 1. Introduction

The triboelectric nanogenerator (TENG) is emerging as an energy-harvesting device that converts mechanical energy into electricity based on a combination of the effects of contact electrification and electrostatic induction [1]. Mechanical energy is one of the most abundant forms of energy that exists in many different forms in our living environment. To harvest these mechanical energies, the concept of environmental friendliness is regarded as one of the most important aspects for the development of a clean and sustainable energy source.

The commonly used materials for the TENG fabrication are polymers, such as poly-dimethylsiloxane (PDMS) [2,3], poly-vinylidenefluoride (PVDF) [4,5], poly-tetrafluoroethylene (PTFE) (or Teflon) [6,7], polyimides (or Kapton) [8,9], and polymethyl methacrylate (PMMA) [10,11]. Most of them are synthetic polymers [12], which have high costs and non-degradable environments. Many efforts have been made to develop biodegradable and environmentally friendly triboelectric materials. These include plant-based materials, such as wood [13], leaves [14], and cellulose [15] and animal-based degradable materials, such as chitosan [16], silk fibroin [17], and gelatin [18].

Natural rubber (NR) is a natural polymer, and its chemical structure is cis-1,4-polyisoprene, which is typically extracted from the tree *Hevea brasiliensis* [19]. Natural rubber latex has been widely used as raw material for manufacturing a wide range of industrial products [20]. The majority of NR products are utilized in kinetic environments that involve motions and vibrations. In this respect, NR is a crucial candidate for biodegradable triboelectric materials to harvest large-scale mechanical energy. Moreover, NR has the feasibility to form composite materials by adding nanoparticles or filler materials and to modify its internal and surface structure to intensify triboelectric charges in order to boost the energy conversion performance of the TENG. Recently, there were a few studies on NR-based TENGs, including silica-based rubber compounds for harvesting mechanical energy from car tires [21], stretchable rubber-based TENGs as self-powered body motion sensors [22], and NR nanocomposite TENGs for energy-harvesting applications [23,24].

Regarding the realization of the practical uses, many approaches have been proposed to enhance the electrical output power of TENGs. Extensive studies have been focusing on the promotion of triboelectric charge quantities in triboelectric materials. This can be done by increasing the surface areas and charge retention abilities or capacitances of the triboelectric materials [25,26]. There are many different ways to modify triboelectric materials for enhancing the power output of the TENG, including surface modification, such as plasma etching [27], micro/nano-patterning [28,29], soft lithography [30], and internal structure modification into porous or sponge structures [31,32,33].

Porous-structured materials have been employed to improve the TENG performance. This contributes to the increased electrification in the internal structure, which promotes triboelectric charge generation and accumulation [34,35]. Activated carbon (AC) is a carbonaceous material with a high porosity and surface area, which can be derived from natural carbon sources, such as plants, animals, and minerals [36]. Human hair is a bio-waste with a high carbon content [37], which is attractive to be used as a starting material for producing activated carbon. With AC’s high specific surface area, ACs derived from human hair (ACH) were found in a variety of applications, such as electrode materials for super-capacitors [38] and batteries [39], gas adsorption [40], and wastewater treatment [41].

In this work, AC derived from human hair was introduced as a filler material for NR, which was employed as a triboelectric material for TENG. This work was the first report on using human bio-waste and natural products to fabricate a biodegradable TENG with a high energy-conversion performance. The effect of ACH filler content in an NR-ACH composite on TENG performance was investigated. The microstructural characterizations of ACH and NR-ACH composites were performed to explain their contribution to the enhancement of energy conversion performance. In addition, the energy-harvesting applications of the fabricated TENG to charge a capacitor and to power a small electronic device and motion sensing application were demonstrated.

## 2. Materials and Methods

### 2.1. Preparation of ACH

Human hair was collected from barber shop; it was initially cut into small pieces with lengths of 2–3 mm. Then the hair was washed with iso-propanol and acetone and dried in oven at 80 °C for 2 h. The dried hair was then pre-carbonized in the presence of Ar at 300 °C for 90 min. The pre–carbonized product was left in 2M KOH (KemAus, New South Wales, Australia) for 48 h and then dried at 80 °C for 6 h. After that, the powders were carbonized at 800 °C for 2 h in an Ar atmosphere. The obtained product was washed with hot DI water and followed by using a 1M HCl (RCI Labscan, Bangkok, Thailand) solution several times to remove any trace of potassium. The activated carbon product was obtained after the product dried overnight at 80 °C.

### 2.2. Preparation of NR-ACH Composite Film

The commercial NR latex used in this work was purchased from the Thai Rubber Latex Group Public Co., Ltd. (Samut Prakan, Thailand) with dry rubber content of 61%. Sodium dodecyl sulfate (SDS, Ajax Finechem, Thai Rubber Latex Group Public Co., Ltd., Samut Prakan, Thailand) was used as a dispersing agent. 10 mL of NR latex was mixed with ACH at 0.3, 0.6, 0.9 wt% and 0.5 mL of 20 mM SDS by using magnetic stirring for 10 min to ensure a homogeneous mix. A total of 2 mL of the mixture was cast on an indium tin oxide (ITO) substrate with an area of 4 × 4 cm^2^ to obtain film thicknesses of approximately 0.5 mm. A set of three specimens was prepared for each experimental condition. The specimens were left dry at room temperature for 2 days and then cured at 80 °C for 6 h. The samples were then ready for TENG performance tests.

### 2.3. Material Characterizations

ACH was characterized by using Raman spectroscopy (SENTERRA, Bruker, Billerica, MA, USA), scanning electron microscopy (SEM, Helios Nanolab, FEI, Lausanne, Switzerland), transmission electron microscopy (TEM, TECNAI G2 20, FEI), and an X-ray diffraction (XRD) analysis (PANalytical EMPYREAN, Malvern, UK). The morphologies and crystal structures of NR-ACH composite films were studied using a SEM and XRD, respectively. Dielectric constants were measured using an impedance analyzer (Keysight E4990A, Santa Rosa, CA, USA) at room temperature.

### 2.4. TENG Fabrication and Output Measurement

**Figure 1:**
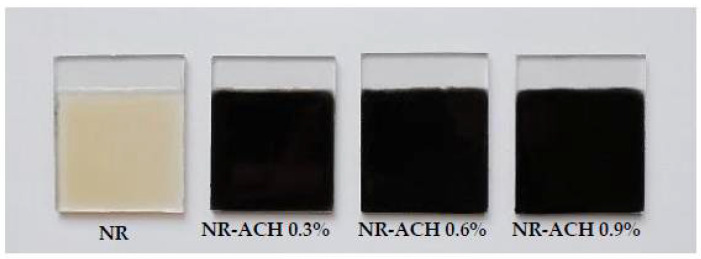
Digital image of the fabricated triboelectric electrodes, including NR and NR-ACH 0.3, 0.6, and 0.9% composites coated on ITO substrates.

**Figure 2:**
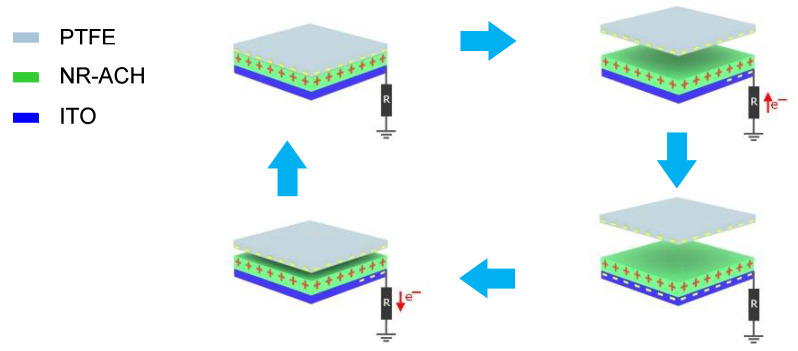
Working mechanism of the NR-ACH TENG.

The TENG was assembled by using an NR-ACH film on ITO glass (Figure 1) as a bottom tribopositive material and a PTFE as a top tribonegative material using a single electrode configuration with the contact area of 4 × 4 cm^2^ as schematically illustrated in Figure 2 and Supplementary Video S1. The energy conversion performance of the fabricated TENG was examined by measuring output voltage and current using an oscilloscope (Tektronix DPO2002B, Tektronix China Ltd, Shanghai, China) and a digital ammeter (Kiethley DMM6500, Tektronix China Ltd, Shang Hai, China), respectively. The TENG performance was tested under a vertical contact-separation mode. The voltages and current output signals were acquired under the mechanical impact force of 10 N with impact frequency of 5 Hz, which was driven by a DC motor (24 V with 2500 rpm maximum speed).

## 3. Results

The triboelectric electrodes fabricated from the NR and NR-ACH composite films coated on ITO substrates as presented in Figure 1 were used as bottom electrodes for the TENG device. The TENG device configuration for performance testing and the TENG’s working mechanism are illustrated in Figure 2. The NR and NR-ACH composite films were tribopositive materials, and a PTFE sheet was used as a paired tribonegative material. The electricity generated upon the contact electrification and electrostatic induction effects are described as follows. When the surfaces of the PTFE and NR-based materials are in contact, surface charges with different signs are formed on the two surfaces; negative surface charges form on the PTFE and positive ones form on the NR-based material. The separation of the two surfaces causes a potential drop, which induces free electrons to flow from the ground to the conductive ITO to balance the potential. The flow of electrons in this state generates a positive current signal. When the two surfaces return to contacting again, the potential drop is reduced and disappears. This causes electrons to flow back to the ground, generating a negative current signal.

**Figure 3:**
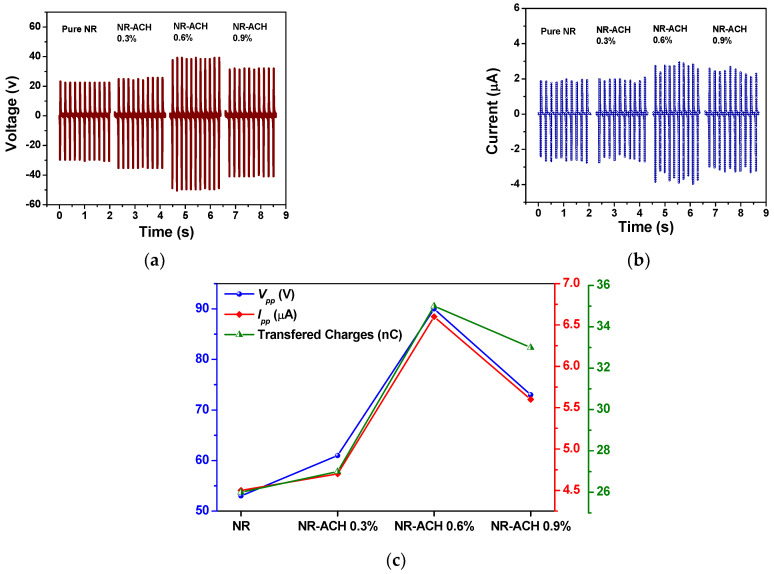
(**a**) Output voltage, (**b**) output current, and (**c**) transferred charges of NR and NR-ACH 0.3, 0.6 and 0.9% TENGs.

The electrical outputs of the NR-ACH TENGs fabricated from NR-ACH composites at ACH concentrations of 0, 0.3, 0.6, 0.9 wt% are presented in Figure 3. The electrical outputs were found to increase with increasing ACH concentrations and reach a maximum peak-to-peak voltage (*V_pp_*) of 90 V and current (*I_pp_*) of 6.6 µA in the NR-ACH 0.6% TENG as shown in Figure 3a,b. These outputs were higher than those of the unmodified NR, which were 53 V and 4.5 µA. However, at the ACH concentration of 0.9%, the electrical outputs of the NR-ACH TENG were dropped. The transferred charges derived from the current output signals of all TENGs are plotted along with *V_pp_* and *I_pp_* as presented in Figure 3c. It was found that the transferred charges of all the fabricated NR-ACH TENGs exhibited the same trend with their voltages and current outputs, and the maximum transferred charges of 35 nC were achieved from the NR-ACH 0.6% TENG.

It was seen that the improvement in TENG performance was due to the presence of ACH. In order to explain the contribution of ACH to the electrical output performance of the TENG, dielectric properties and microstructural characterizations were performed and described in the following section.

**Figure 4:**
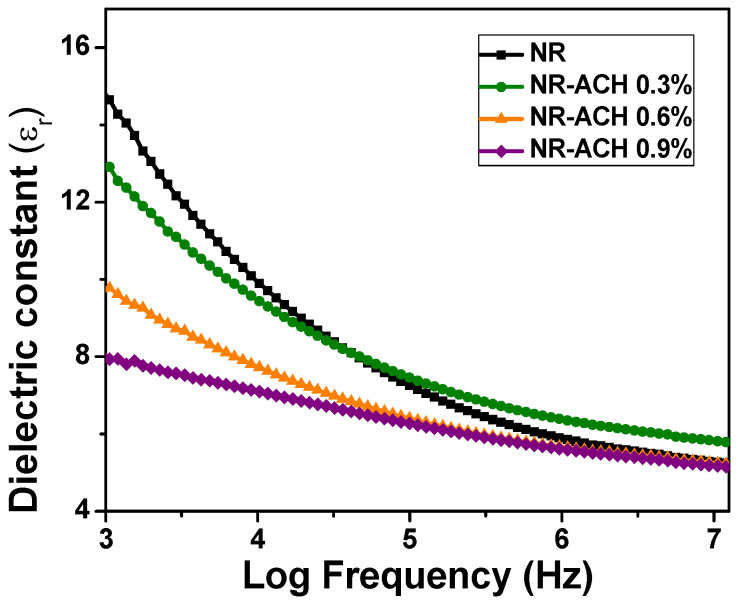
Dielectric constant of NR and NR-ACH 0.3, 0.6, and 0.9% composite films.

The dielectric constants of the NR-ACH composites were probed. The plots of dielectric constants as functions of electric field frequency are shown in Figure 4. It was found that the addition of ACH with increasing concentrations resulted in the reduction of the dielectric constants of the NR composites. This indicated that the percolation point (*f_c_*) of the conductive filler material was achieved, leading to the increased electrical conductivity of the composites. This also suggested that ACH was electrically conductive. The electrical conductivity of the composite (*σ_c_*) is proportional to the filler conductivity (*σ_fil_*) and filler concentration (*f*), which is described by the following expression,

(1) $$\sigma_{c}\propto \sigma_{fil}{\left(f-f_{c}\right)}^{t}$$

where *t* is the critical exponent (>1.0) [42].

### 3.1. Microstructural Characterization

**Figure 5:**
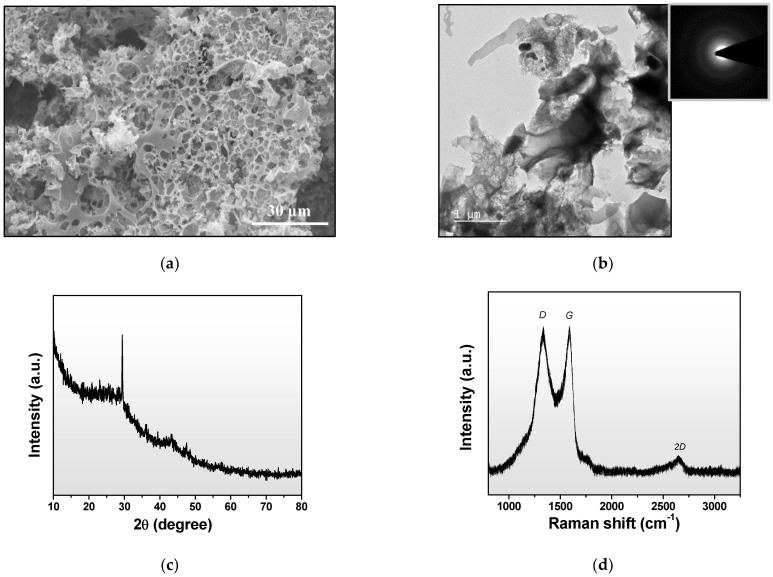
(**a**) SEM image, (**b**) TEM image, (**c**) XRD pattern, and (**d**) Raman spectrum of activated carbon derived from human hair (ACH).

The microstructures and morphologies of the synthesized ACH were investigated. The SEM image in Figure 5a revealed that the ACH had a predominantly porous structure with a relatively large pore size. The TEM image in Figure 5b showed nanosheet structures distributed across the porous structure, and the inset selected area diffraction (SAED) suggested that ACH has an amorphous structure. The XRD pattern of the as-received ACH in Figure 5c showed two broad peaks at around 23° and 43°, which corresponded to the (002) and (100) planes of amorphous carbon, which were similar to those in previously reported activated carbons derived from human hair [38] and any other activated carbons [43].

Raman spectroscopy was performed to investigate the structures of carbon materials, which are presented in Figure 5d. The peaks observed at wave numbers of 1330, 1583, and 2662 cm^−1^ are called D, G, and 2D bands, respectively. The intensity of the G band indicates the presence of sp^2^ carbon in a graphitic structure, and that of D band corresponds to the vibrations of disorder sp^3^ carbon. The I_G_/I_D_ indicates the graphitic degree of carbon material. This suggested that the ACH contained a reasonable amount of sp^2^ carbon. The presence of sp^2^ carbon accounts for the electrical conductivity of carbon materials. This contributes to the reduction of the dielectric constant when adding increasing amounts of ACH in NR composites.

The improved electrical output of the TENG was ascribed to the presence of porous ACH that had a high surface area [44] and the additional free electrons of the sp^2^ carbon structure, which acted as charge trapping sites for the generation of triboelectric charges during electrification events [3,45,46]. The enhancement of triboelectric charge density (*σ*) gave rise to the improved TENG electrical outputs. For the contact-mode TENG, the open-circuit voltage (*V_oc_*) and short-circuit current (*I_sc_*) are given by using Equations (2) and (3), respectively [47]:

(2) $$V_{oc}=\frac{\sigma x\left(t\right)}{\varepsilon_{0}}$$

(3) $$I_{sc}=\frac{S\sigma d_{0}v\left(t\right)}{{\left(d_{0}+x\left(t\right)\right)}^{2}}$$

where *S*, *x*(*t*), *v*(*t*), *d_0_*, and *ε_0_* are the contact area size, separation distance, contact electrode velocity, effective thickness constant, and electrical permittivity of free space, respectively.

**Figure 6:**
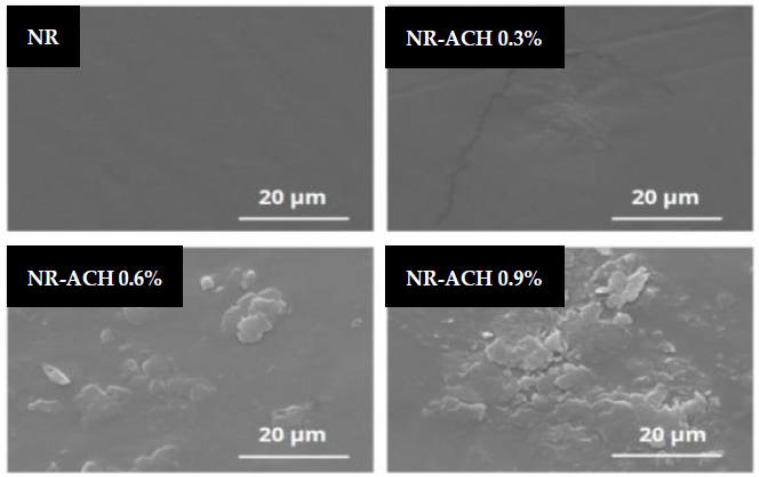
SEM surface images of NR and NR-ACH composite films with ACH concentrations of 0.3%, 0.6%, and 0.9%.

Generally, the TENG performance can be improved by magnifying the triboelectric charge density, which can be done by increasing the size of the contact area and the dielectric constant of triboelectric materials. However, our results showed that the TENG output increased while the dielectric constant decreased with increasing ACH concentrations. This suggested that contact area played a major role in controlling TENG performance in our case. The surface morphologies of the NR and NR-ACH composite films were therefore investigated by using SEM imaging as displayed in Figure 6. The surface morphologies of the composite films changed with the increasing ACH content, as seen by the increasing number of particles observed, which was different from the plain NR, which had a surface that was flat and clean without any particles observed. It was noticed that a high ACH content in the NR-ACH 0.9% caused a predominant presence of ACH on the film surface, and the agglomeration of the ACH was observed. This change in surface morphology adversely affected the TENG output performance.

### 3.2. TENG Working Condition and Applications

**Figure 7:**
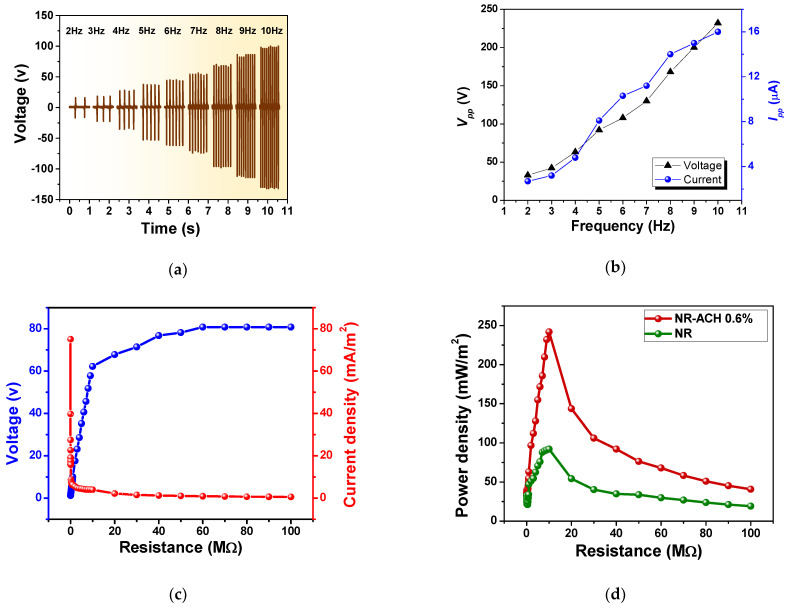
(**a**) Frequency dependence of electrical output voltage and (**b**) the plot of output voltages and currents versus working frequencies ranging from 2–10 Hz of the NR-ACH 0.6% TENG. (**c**) The plot of the measured voltage and current and (**d**) the corresponding power density of the NR-ACH 0.6% TENG when connected at various load resistances.

The TENG performance was found to be dependent on the operation frequency as shown in Figure 7a. The *V_pp_* and *I_pp_* increased with increasing working frequencies from 33 V and 3 µA at 2 Hz to 232 V and 16 µA at 10 Hz. The relation of *V_pp_* and *I_pp_* as a function of working frequencies was plotted as presented in Figure 7b. The increased TENG output with working frequency was attributed to the short contact-separation cycle leading to the retention and accumulation of tribo-charges on the surfaces [48].

The electrical power of the fabricated NR-ACH TENG was measured to determine the highest electrical energy that delivered to an external load. The power output measurement was performed under an applied mechanical force of 10 N at a 5 Hz frequency. The plots of output voltages and currents measured at various load resistances and the delivered power densities are shown in Figure 7c,d, respectively. The highest power density of 242 mW/m^2^ was achieved at the matched load resistance of 10 MΩ, which was almost three times higher than that of the plain NR TENG (92 mW/m^2^). In addition, the power density reported in this work was larger than that of other biodegradable TENGs, such as bacterial nanocellulose TENGs (4.8 mW/m^2^) [49], wood-based TENGs (57 mW/m^2^) [13], chitosan TENGs (15.7 mW/m^2^) [50], and the stretchable TENGs made from a conductive composite and an elastomer (23 mW/m^2^) [51].

**Figure 8:**
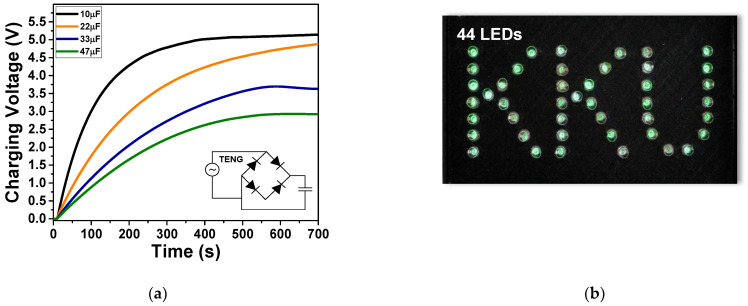
(**a**) Charging voltage profiles of capacitors charged by the electricity generated from fabricated TENG; (**b**) the TENG electrical output can instantaneously light up 44 green LEDs.

**Figure 9:**
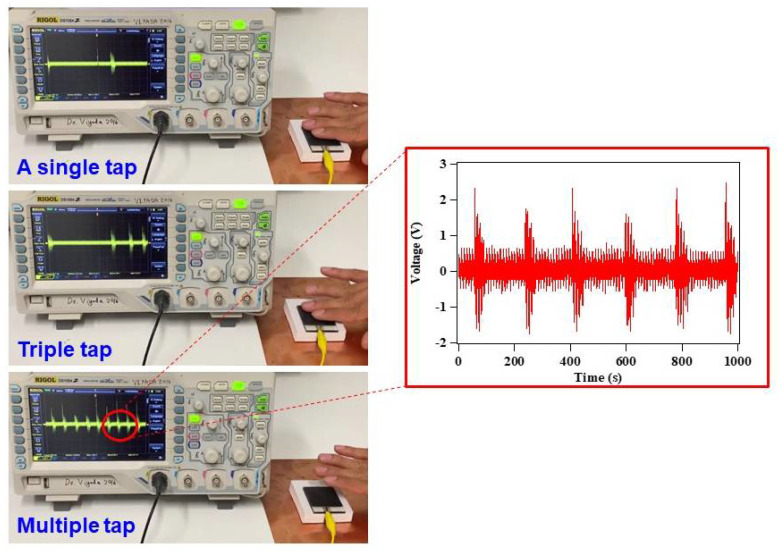
The demonstration of motion sensing application of the fabricated NR-ACH TENG under a single electrode configuration using finger taps and the inset of the generated voltage signal.

The electricity generated by the NR-ACH TENG was able to be charged or stored in commercial capacitances as illustrated by the voltage charging profiles of the 10, 22, 33, and 47 µF capacitors in Figure 8a. The 47 µF capacitor was charged to 3 V in 550 s. In addition, the electrical power was demonstrated to instantaneously light up 44 green LEDs as shown in Figure 8b and Video S1. Furthermore, the motion sensing application of the NR-ACH TENG was demonstrated. A single-electrode mode TENG was tested using bare fingers as contact electrodes as presented in Figure 9 and the Video S2 in Supplementary Material. The electrical signal was instantaneously generated when the fingers touched the NR-ACH surface, which is shown in Figure 9. This signal can be applied as a motion sensor that can detect any movement, such as that of the human body or an object on the film surface.

## 4. Conclusions

The biodegradable NR-ACH composite was synthesized and used to fabricate a TENG to convert mechanical energy into electricity. The addition of ACH was found to improve the electrical output performance of the TENG due to the high surface areas of the porous structures of ACH filler materials, which also acted as charge trapping sites to intensify triboelectric charges generated during electrification events. The NR-ACH TENG with the optimum ACH concentration of 0.6% generated the highest electrical power density of 242 mW/m^2^, which was almost three times larger than that of the unmodified NR TENG. The generated electrical power was able to charge the commercial capacitors to power small electronic devices. In addition, the NR-ACH TENG, with a single electrode configuration, was able to detect the movement of the human body, which could be applied to a motion-sensing application.

## Data Availability

The data presented in this study are available in the article.

## Supplementary Materials

The following supporting information can be downloaded at: https://www.mdpi.com/article/10.3390/polym14061110/s1, Video S1: Lighting up green LEDs by using the TENG, Video S2: Motion sensing application.
